# Inequalities in Enrollment in Nepal’s National Health Insurance Program: An Intersectional Analysis of Nepal Demographic and Health Survey 2022

**DOI:** 10.3390/ijerph23040521

**Published:** 2026-04-17

**Authors:** Geha Nath Khanal, Kiran Acharya

**Affiliations:** 1 School of Nursing, Midwifery, Allied and Public Health, Canterbury Christ Church University, Canterbury CT1 1QU, UK; 2 New ERA, Rudramati Marga, Kalopul, Kathmandu 44600, Nepal; acharya.kiran1@gmail.com

**Keywords:** health insurance, health inequality, intersectionality, Nepal, universal health coverage

## Abstract

**Highlights:**

**Public health relevance—How does this work relate to a public health issue?**

- National health insurance program (NHIP) of Nepal experiences several dimensions of inequality.
- It provides evidence for targeted policy interventions to increase enrollment in NHIP.

**Public health significance—Why is this work of significance to public health?**

- This study constructs a composite measure of intersectional disadvantage in NHIP enrollment.
- The use of composite indicators of intersectional disadvantages (wealth, education, gender and ethnicity) shows how multiple markers interact to create deeper inequality.

**Public health implications—What are the key implications or messages for practitioners, policy makers and/or researchers in public health?**

- Intersectional disadvantages create compounded barriers to NHIP enrollment.
- Targeted interventions addressing intersection of wealth, ethnicity, gender, and education are necessary to reduce inequality.

**Abstract:**

Nepal’s National Health Insurance Program (NHIP), launched in 2016, continues to show low enrollment rates and substantial socio-economic and geographical inequalities hinder the progress towards universal health coverage (UHC). This study uses a composite indicator of intersectional disadvantages to examine how multiple equity markers (wealth quintile, education status and ethnicity) interact to shape inequalities in NHIP coverage. Data were drawn from the nationally representative 2022 Nepal Demographic and Health Survey. Key predictors are wealth status, education, ethnicity, residence, province, ecological zone and marginalization status. A composite measure of intersectional disadvantage was constructed using three socioeconomic dimensions: wealth, education, and ethnicity. Binary logistic regression, concentration indices, and concentration curves were used to assess the patterns of inequality in NHIP coverage. Results show that only 10.2% of men and 10.8% of women were enrolled in the NHIP. Enrollment varied markedly by province, with highest in Koshi (21.8% for men and 22.9% for women) and lowest in Madhesh (3.1% for men and 2.7% for women). Enrollment was disproportionately higher among wealthier, more educated, and ethnically advantaged groups. This disparity is starkest for those with an intersection of triple disadvantage (poor, illiterate, and disadvantaged ethnicity) and had substantially lower coverage (3.0% for men and 3.4% for women) compared to those facing no disadvantage (18.4% for men and 22.9% women). The concentration curve analysis confirmed that wealthier women and men had greater access to NHIP. Multivariable analysis showed that women and men with no disadvantages were more likely to be enrolled in NHIP than individuals in triple-disadvantage groups. These findings highlight persistent inequities in NHIP, which undermine its contribution to financial risk protection. Targeted interventions are urgently required, including effective implementation of existing subsidies for poor households, expansion of health facility networks in underserved provinces like Madhesh, and tailored outreach programs that address the intersection of ethnicity, wealth, and education in both genders to accelerate equitable progress towards UHC.

## 1. Introduction

Universal Health Coverage (UHC) has become a central driver of the global health agenda, representing an important long-term global healthcare priority. As a key mechanism for advancing equity, health outcomes, financial protection, and economic development, UHC ensures all people can access quality, affordable healthcare services when needed [1,2]. Social health protection programs have become a prominent strategy for achieving UHC in low- and middle-income countries (LMICs) to reduce catastrophic out-of-pocket expenditures (OOPE) and improve healthcare access [3].

Nepal implemented the National Health Insurance Program (NHIP) in 2016 as a pilot in three districts: Kailali, Baglung and Ilam [4,5]. The program was gradually expanded in a phased manner and had reached all the 77 districts and 750 of the 753 local governments by March 2026 [6]. The program considers families (up to five members) as a unit for enrollment. The base insurance premium for a family is set at NPR 3500 per year (1 USD = NPR 149), with an additional charge of NPR 700 for each additional member. The benefit package provides coverage up to NPR 100,000 for the family and NPR 20,000 for each additional member with the maximum ceiling of up to NPR 200,000 per family [7]. The benefit package includes 1289 types of medicines [8]. As of March 2026, Health Insurance Board (HIB), the public institution responsible for operating and managing the NHIP has empaneled 425 health facilities as first service points (FSPs) and 85 referral hospitals [6,9]. The FSPs are designated health facilities like hospitals or primary healthcare centers (PHCs) that individuals must select during their enrollment. They can select only the public health facilities as FSPs during enrollment. Insured individuals need to visit the FSP that they had chosen during enrollment for routine check-ups, while they may visit the referral hospitals upon referral from FSPs or in the case of an emergency [5]. Out of 29.1 million population, 98.7 million population have been enrolled in the scheme. The active enrolled population is about 60% only (5.97 million) [6].

Some studies from Nepal using Demographic and Health Surveys (DHS) have examined how multiple forms of disadvantages (or advantage) interact to influence access to healthcare [10]. Although national representative studies on health insurance have explored individual dimension of disadvantage such as wealth, ethnicity, or educational attainment in relation to health insurance enrollment [11,12,13,14,15], they have not applied the intersectionality lens in assessing the disparity in health insurance enrollment. Intersectionality provides a valuable framework. It helps to examine how overlapping social identities intersect and create unique experiences of advantages and disadvantages [16,17,18]. For example, the barrier to health insurance faced by poor women differs from those faced by women who are poor, illiterate and from a disadvantaged ethnic group. Understanding this compounded barrier is essential for effective implementation of health insurance policy in Nepal.

Nepal’s health insurance policy seeks to achieve UHC and improve access to healthcare to general public [19]. It also aims to provide subsidies for targeted population including the poor households [20]. However, such blanket policy approach may fail to reach the most marginalized populations. Other social factors like ethnicity and education often create extra layer of disadvantage. Current policies have yet used intersectionality to identify and support the most vulnerable segments of population within the poor households. In contrast to previous studies, which examined socio-economic inequality of health insurance in isolation, this study provides novelty in literature. It applies intersectionality framework to quantify how multiple disadvantages (being poor, illiterate and in a disadvantaged ethnic group) affects NHIP enrollment in Nepal. This approach provides clear empirical evidence. It shows the need for targeted strategies to reach people facing multiple overlapping disadvantages in society. Furthermore, this research directly supports the “leave no one behind” principle. This is a core commitment of the Sustainable Development Goals (SDGs) by identifying the most marginalized groups in access to health insurance coverage [21]. It also aligns SDG goals 10, which aim to reduce inequalities. The findings offer important insights for assessing and addressing persistent disparities in health equity [22].

## 2. Materials and Methods

### 2.1. Sampling Design and Data Source

We used secondary data from Nepal Demographic and Health Survey (NDHS) 2022 which is a nationally representative cross-sectional survey. NDHS 2022 is the sixth round periodic nationally representative survey conducted every five years. It is also the first survey conducted after the NHIP was implemented across Nepal [23]. The survey used a two-stage stratified sampling method. Each province was stratified into urban and rural areas. Detailed information on the sampling design and survey methodology appears in the final report [23].

A total of 4913 men and 14,845 women aged 15–49 were interviewed face-to-face by trained interviewers using structured questionnaires. All eligible women aged 15–49 in selected households were interviewed. Men aged 15–49 were interviewed only in half of the selected households. This approach, interviewing all women and half men is a standard practice in DHS conducted in over 90 countries [24]. Previous studies have also analyzed women and men separately [12,13,14,15,25]. The flowchart showing women and men distribution for this study is shown in File S1 (Figure S1).

### 2.2. Outcome Measures

The primary outcome of this study was enrollment in NHIP. We assessed this separately for women and men aged 15–49 years. Both women and men were asked: ‘Are you covered by any health insurance? If yes, what type of health insurance are you covered by?’ We classified responses that mentioned government health insurance as NHIP enrollment.

Independent variables included socioeconomic and geographic characteristics such as ethnicity, wealth quintile, education status, place of residence, province, ecological region and marginalization status. For this study, we merged six categories of ethnic groups into two groups: disadvantaged ethnicities (Dalits, Muslims, Terai ethnic groups, and disadvantaged Janajatis) and advantaged ethnicities (Brahmins/Chhetris and relatively advantaged Janajatis). We dichotomized education into illiterate (unable to read and write) and literate (able to read and write and having completed at least primary education).

We also dichotomized wealth quintiles into two groups: poor (lower 40% or lowest two quintiles) rich (upper 60% or higher three quintiles). This binary classification aligns with established literature documenting historical and structural patterns of privilege and exclusion in Nepal by ethnicity, wealth and education [10]. We constructed a composite disadvantage measure using education status, wealth quintile, and ethnicity to examine intersectional inequalities. This dichotomization was done primarily to address small sample sizes when combining multiple dimensions of disadvantage. We then combined these dichotomous variables to form a composite marginalization status variable. The eight original categories of marginalization status were collapsed into four categories, based on the number of concurrent disadvantages a woman experienced: triple, double, single, or no disadvantages [10]. This approach operates intersectionality. It captures the cumulative burden of multiple marginalized identities. It also allows testing whether health insurance coverage decreased significantly as the number of disadvantages increased.

We used an additive approach (single, double, and triple disadvantage) to apply intersectionality in a quantitative framework. This method helps us examine how overlapping social disadvantages compound inequalities in health outcomes. It is widely used in public health research with survey data. The detailed definition of all the variables is explained in File S1 (Table S1).

### 2.3. Statistical Analysis

Results were disaggregated by sex, and an intersectional analysis was performed to examine inequalities across wealth quintiles, education status, and ethnicity. Categorical variables were summarized as frequencies, percentages (%), and 95% confidence intervals (CIs) across the relevant associated variables which are presented in File S1 (Table S2).

We used bivariate and multivariable binary logistic regression analyses to estimate the adjusted effects of NHIP enrollment separately for women and men. We reported both unadjusted odds ratio (uOR) and adjusted odds ratios (aOR) to show the magnitude of association relative to the reference category.

Statistical significance was determined at a *p*-value < 0.05, with 95% confidence intervals used to assess precision. We checked the multicollinearity among covariates using the Variance Inflation Factor (VIF). A cutoff value of 10 was applied before running the multivariable models.

Further, we also assessed model fit using Stata’s postestimation goodness-of-fit test, which indicated that the model adequately fitted the data. The level of statistical significance was set to 0.05. Our main objective was to examine inequalities using wealth quintile, education status, and ethnicity as intersectional dimensions of marginalization. Therefore, these were the primary predictors in the models. Other important variables, such as health status, prior service utilization, and disability, are reported in File S1 (Table S3).

The Erreygers normalized concentration index (ENCI) was used to quantify socio-economic inequalities in NHIP enrollment [26]. The ENCI is suitable for bounded variables, such as dichotomous measure of enrollment in NHIP (Yes = 1 and No = 0) [26,27,28]. The ENCI is computed using the following equation:

$$E(C)=\frac{4\mu }{b-a}\times C$$

where

μ is the mean of the health variable (NHIP enrollment);
a and b are lower and upper bounds of the variable;
C is the traditional concentration index.

$$C=\frac{2}{\mu }\;Cov(y,R)$$

where

y is the health variable.
μ is the mean of the health variable (NHIP enrollment).
R is the fractional rank of individuals in the distribution of SES.
Cov denotes the covariance between y and R.

The ENCI ranges between −1 and +1. A value of zero indicates no socioeconomic-related inequality in the health outcome, a positive value indicates that the outcome is more concentrated among individuals with higher socioeconomic status (pro-rich inequality), and a negative value indicates that the outcome is more concentrated among individuals with lower socioeconomic status (pro-poor inequality). This normalization is widely used in studies examining socioeconomic inequalities in health outcomes. The ENCI and standard error (SE) is presented in the result section.

The ‘svyset’ command in Stata (MP version 17.0) was used to apply sampling weights and account for the complex survey design (separately women and men), ensuring unbiased estimates for data management and statistical analysis [29], and figures were prepared using both Stata and R (version 4.4.3) [30]. The gender imbalance in sample sizes is by design in DHS surveys and does not compromise the validity of our gender-specific analyses—particularly since we applied complex survey analysis and gender-specific weights.

## 3. Results

### 3.1. Descriptive Summary

The descriptive analysis included 4913 men and 14,845 women. The enrollment for men was 10.2% [95% CI: 8.8–11.8] and that of females was 10.8% [95% CI: 9.6–12.2] respectively (Table 1). The enrollment was consistently higher among older, more educated, and wealthier individuals in both genders. Clear disparities were observed across ethnicities and provinces. Enrollment was also higher among internet users and those with media exposure (radio, television or newspapers). Tobacco use was linked to lower enrollment. Several other variables showed no clear association. Details of descriptive analysis are in File S1 (Table S2).

### 3.2. Measurement of Socio-Economic Inequality

Structural factors showed significant association with coverage. Ethnic disparities were clear: advantaged ethnic men had 15.8% coverage compared to 7.5% among disadvantaged ethnic men. This pattern was similar for women (advantaged: 17.8% vs. disadvantaged: 7.3%).

Education also revealed large inequalities. Literate men had 10.9% enrollment versus 2.5% enrollment for illiterate men. Among women, the figures were 13.0% versus 4.5%. Wealth also showed strong gradient with enrollment. The wealthiest men (top 60%) had 12.0% coverage compared to 6.8% among poor men (bottom 40%). A similar trend appeared among women (rich:13.7% versus poor: 5.8%).

At the intermediatory level, provincial inequalities were observed. Koshi province had highest enrollment (men: 21.8% and women: 20.4%) while Madhesh Province recorded the lowest (men: 3.1% and women: 2.7%). Urban residence was linked to higher coverage only among women (urban: 12.2% versus rural: 7.8%), while the difference was not significant for men (urban: 11.0% versus rural: 8.4%). Ecological zone (Mountain, Hill, Terai) showed no significant association with NHIP coverage for either gender.

Intersectional analysis revealed compounded disadvantages. Individuals with triple disadvantage (poor, illiterate, and disadvantaged ethnicity) had the lowest coverage (men: 3.0%; women: 3.4%), while those with no disadvantage (rich, literate, and advantaged ethnicity groups) had the highest rate (men: 18.4%; women: 22.9%).

**Figure 1:**
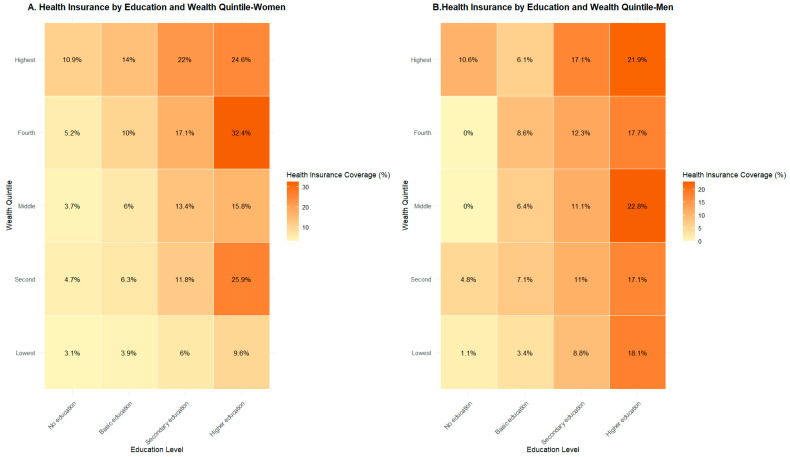
Enrollment of health insurance among women (**A**) and men (**B**) in Nepal by education status and wealth quintile.

Figure 1 shows clear inequalities in NHIP enrollment by education level and wealth quintile. Enrollment increases with higher education and higher wealth levels for both genders. Higher educated women in the fourth wealth quintile recorded the highest enrollment (32.4%), compared to only 3.1% among uneducated women in poorest quintile. Similarly, higher educated men in third and fifth wealth quintiles had the enrollment rate of nearly 22%, while uneducated men in poorer groups showed almost-zero enrollment (0% in third and fourth quintile and just 1.1% for lowest quintile).

**Figure 2:**
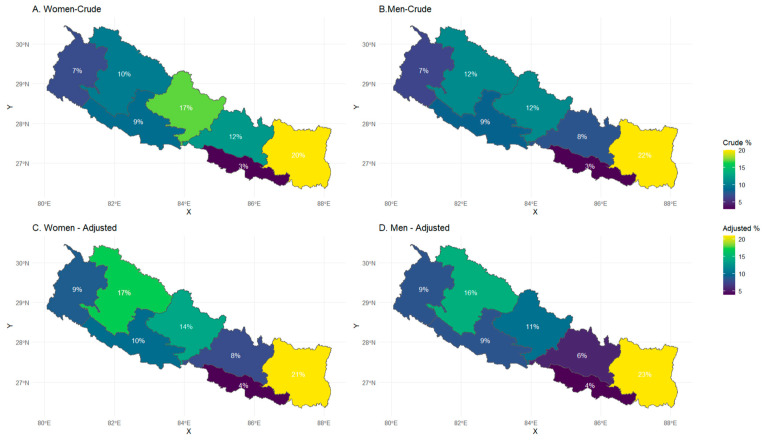
Health insurance enrollment (crude and adjusted) by province in Nepal.

Figure 2 presents provincial variations in NHIP enrollment. Both crude rates (Panel A: Women; Panel B: Men) and adjusted rates (Panels C: Women; Panel D: Men) shows Koshi province maintains the highest NHIP enrollment for both genders, while Madhesh consistently ranks the lowest rates. These patterns highlight significant geographic inequalities in Nepal’s health insurance uptake. Adjustments were made using three key covariates: age, education, and wealth quintile.

### 3.3. Results from Measures of Inequality by Geographical Location

**Figure 3:**
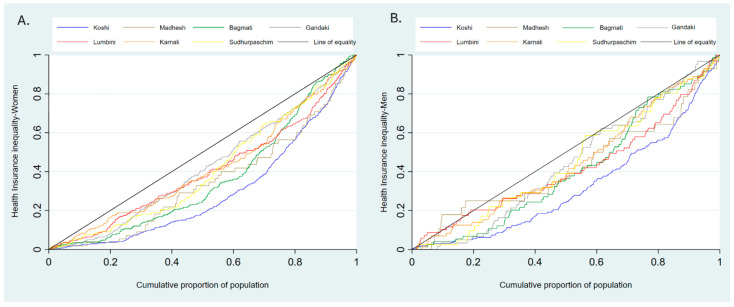
Concentration curve showing wealth-related inequality in NHIP enrollment in women (**A**) and men (**B**) by provinces.

Figure 3 shows inequality in health insurance enrollment in provinces by wealth status. The concentration curve lies below the line of equality. This indicates pro-rich inequalities in NHIP enrollment across seven provinces for both genders. Women from Koshi and Bagmati province showed steeper curves, which shows a stronger pro-rich enrollment bias. In contrast, Sudhurpaschim province showed relatively equitable enrollment. Among men, Koshi again showed the highest wealth-related inequality. These patterns confirm systemic structural advantages for wealthier households in NHIP enrollment.

### 3.4. Results from Measures of Inequality by Marginalization Status

**Figure 4:**
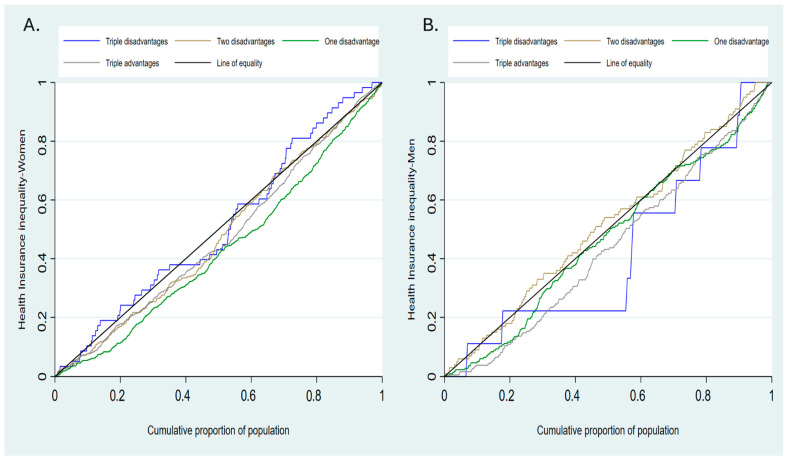
Concentration curve showing wealth-related inequality in NHIP enrollment in women (**A**) and men (**B**) by marginalization status.

Figure 4 below shows the concentration curve in health insurance enrollment among women and men by their marginalization status. The findings show that NHIP coverage is most concentrated among advantaged groups.

### 3.5. Socioeconomic and Education-Based Relative Inequality in the Enrollment of NHIP

Table 2 presents the ENCI and summarizes the magnitude of socioeconomic and education-based inequalities. Positive ENCI values mean that NHIP enrollment is more concentrated among higher wealth quintiles and more educated groups. Women experienced greater socioeconomic inequality (ENCI = 0.102) than men (ENCI = 0.071). This pattern is observed in both rural (women: 0.077 vs men: 0.060) and urban areas (women: 0.104 vs men: 0.063). Socioeconomic inequality varies across ecological zones. The strongest pro-rich bias appeared in the Hill zone for both men (ENCI = 0.086) and women (ENCI = 0.129). Provincial differences are also clear. All provinces showed socioeconomic inequality among women, with Koshi showing the highest (0.314) and Madhesh showing lowest (0.026). Among men, disparities were not statistically significant in Madhesh, Gandaki, Karnali, and Sudhurpaschim provinces.

Intersectional analysis revealed more pronounced disparities. Statistically significant ENCI values appeared for both single-disadvantage groups (women:0.053 vs men: 0.027) and no disadvantage groups (women:0.068 vs men: 0.079). These findings highlight persistent socioeconomic, educational, and marginalization-related disparities in NHIP enrollment. The gaps particularly affect women, residents of certain ecological zones, and specific provinces. Education-based inequality follows a similar pattern, with women generally experiencing higher disparities across zones.

### 3.6. Factors Associated with NHIP Enrollment Among Women and Men

Table 3 indicates that socio-economic disadvantage greatly influences NHIP coverage for both men and women. Adjusted results show that men without disadvantage had approximately 6.4 times greater odds of being covered, while women had about 8 times greater odds compared to those experiencing triple disadvantage. This highlights a clear trend: as marginalization decreases, the likelihood of enrollment rises. Provincial differences were notable, with all provinces showing lower coverage odds compared to Koshi; Madhesh had the lowest odds for both genders (about 90% lower). For women, urban residence was linked to 30% lower odds of coverage than rural residence, whereas this relationship was not statistically significant for men. Ecological zone did not significantly affect NHIP coverage for either gender. Further multivariable analysis details are available in File S1 (Table S3).

## 4. Discussion

This study examined the coverage of NHIP among women and men using nationally representative survey data. Overall enrollment was low: 10.2% for men (95% CI: 8.8, 11.8) and 10.8% for women (95% CI: 9.6, 12.2). This low enrollment highlights a major barrier in achieving universal coverage of NHIP enrollment.

Our results show that advantaged ethnic groups, literate individuals, those in higher wealth quintiles, people living in rural areas, and residents of Koshi province had significantly higher coverage than other counterparts. Education strongly predicts NHIP enrollment for both genders. Literate individuals had significantly higher rates than illiterate ones (men: 10.9% vs. 2.5%; women: 13.0% vs. 4.5%). Multivariable analysis confirmed this association (Table 2). These findings suggest that education enhances awareness of NHIP benefits and financial risks of remaining uninsured. This finding aligns with previous studies from Nepal [11,31,32], Ghana [33,34], Namibia [35], Ethiopia [36] and other LMICs [37].

To address this gap, enrollment assistants (EAs) have been recruited at ward level to visit homes, explain the scheme, enroll households and collect premium [5]. These EAs could do more through community outreach. Other effective interventions include setting up information desks or enrollment support booths in the health facilities. School could also facilitate by adding insurance education to the school curriculum or conducting sessions within school health programs [38].

Wealth status showed a clear pro-rich pattern. Wealthier households had higher enrollment compared to poorer ones. This is likely because richer households can more easily afford the premium, while poorest face financial barriers. These findings are consistent with research from Nepal [11,39,40], Ghana [33,34,41,42,43,44,45], Ethiopia [36], Namibia [35] and Nigeria [46]. Ethnicity also played a significant role. Advantaged groups are enrolled at higher rates than disadvantaged ones for both sexes. This echoes with previous Nepalese studies showing higher enrollment among advantaged ethnic groups compared to marginalized groups such as Dalits [11,39]. Geographic disparities were clearly visible. Households in Koshi Province were more likely to enroll than those in the other six provinces, while Madhesh had the lowest rate for both genders. Likewise, recent data shows that although the program has been implemented in 750 local governments, almost half (368) of the local governments do not have any FSPs [6], which might have contributed to low enrollment.

Regression analysis confirmed that all the other provinces had significantly lower odds of NHIP enrollment compared to Koshi Province, and aligns with other previous studies [11]. Lower enrollment in Madhesh, Sudhurpaschim, and Karnali provinces may partly reflect their Human Development Index (HDI) scores, which are below the national average of 0.602 [47]. Analysis of service utilization and empaneled health facility data (File S1, Table S4) suggests another issue: limited access to tertiary or high-quality health facilities offering NHIP services. Enrollment was comparatively higher in districts where tertiary-level facilities were available even in low HDI areas like Jumla district.

Structural barriers also played a role in Madhesh Province. Six of its eight districts rank among the nine districts nationwide with the lowest density of NHIP empaneled facilities (<1 facility per 100,000 population). Four of the top five districts with the lowest enrollment rates (Sarlahi, Dhanusha, Mahottari, and Bara) are in Madhesh Province (File S1, Table S5). This uneven distribution of empaneled health facilities might have reduced access to services and, ultimately, the enrollment which has been analyzed and discussed in File S1 (Analysis S1). In addition, many people in Madhesh Province seek healthcare across the border in neighboring India, where the NHIP card is not accepted. This may further discourage enrollment.

Concentration curves and indices showed that NHIP enrollment was more concentrated among wealthier households for both sexes across all seven provinces. Wealthier households were therefore better protected from financial risk, while poorer ones remained exposed to financial hardship. Studies from Sub-Saharan Africa [48], Nepal [11] and Kenya [49] have demonstrated similar pattern.

To address this issue, the Government of Nepal has a policy to subsidize enrollment for poor households [19,20]. Subsidies are provided to targeted populations including ultra-poor households, senior citizens, female-community health volunteers (FCHVs), those with null disability, leprosy patients, individuals with multi-drug resistance tuberculosis and people living with HIV [20]. However, very few poor households have joined the schemes. The recent data shows that of the enrolled 5.97 million active enrolled population, only 1.63 million are from targeted population [6]. Of these, 75% (1.23 million) are senior citizens [6]. Although more than one in five of the (17.4%) populations is poor, as per multi-dimensional poverty index [50], the program has not delivered subsidies to the poor sufficiently. Weak systems for identifying eligible poor households and lack of clear guidelines remain major problem for this issue [5].

The intersectional analysis illustrated compounded disadvantages. Individuals with triple disadvantage (poor, illiterate, and from a disadvantaged ethnic group) had the lowest (men: 3.0%; women: 3.4%), while those with no disadvantage had the highest enrollment rate (men: 18.4%; women: 22.9%). An intersectionality theory suggests that multiple forms of marginalizations interact to lower the odds of enrollment among the most disadvantaged groups [18,51]. In the adjusted model, having no disadvantage was linked to 6.4 times higher odds for men and 8.0 times for women compared to triple disadvantage. A systematic literature review revealed that vulnerable populations—including individuals with disabilities, female-headed households, ethnic minorities, and displaced persons—have lower enrollments [52].

The ENCI shows persistent socio-economic and education-based inequality in NHIP enrollment. These inequalities favor richer and educated groups and were stronger among women. This suggests structural barriers, such as limited access to information, financial autonomy, or decision-making power within households, may disproportionately affect women’s ability to enroll in the NHIP. The ENCI findings confirm that inequality in NHIP enrollment undermines the progress towards UHC in Nepal. The persistent barriers for poorer, less educated, rural and female population and unequal expansion in Madhesh, Karnali and Sudhurpaschim province compared to Koshi Province indicates several policy and programmatic reforms.

### 4.1. Program and Policy Implications

This study reveals several actionable policy implications to enhance NHIP enrollment and equity, advancing the pathway to UHC goals. First, facing triple disadvantage need targeted approaches based on the intersectionality framework [53]. The policymakers should identify most marginalized and design-targeted interventions. This could include targeted awareness campaigns, integrated outreach programs that links health insurance with other health programs, and shift from blanket subsidies to multi-dimensional targeting that addresses compounded barriers [54].

Second, community outreach programs should merge health insurance awareness with existing community platforms. Subsidies need to move from universal to targeted support for vulnerable populations, and educational initiatives should foster insurance literacy through schools and community mobilizers. Rwanda and Ghana successfully increased enrollment through community-based campaigns delivered through school [55,56].

Third, large provincial disparities—the highest in Koshi and the lowest in Madhesh—highlight the need to improve geographic access. Increasing the number of empaneled health facilities and first service points (FSPs) in underserved provinces could enhance access and uptake. The evidence from Rwanda shows that better healthcare access increases enrollment [55].

Fourth, observed pro-rich bias calls for a stronger system to identify and subsidize poor households. Although subsidies policy exists, the existing mechanisms are weak for its effective implementation [5,7]. Implementing a more robust identification system such as integrating local government data or community-based targeting would help to reach appropriate poor households [5,7]. Ethiopia’s hybrid model, which combined poverty-targeted social protection with health insurance program can be a useful example [57].

Finally, strategic expansion of empaneled facilities is needed in under-performing districts. Policymakers should carefully assess the location of health facilities considering the population distribution and reliable transportation networks to promote equitable access to services.

### 4.2. Strength and Limitation

Our study helps in filling the knowledge gaps in inequality in NHIP enrollment through three methodological advancements. First, it used a large, national representatives survey, which supports generalizability to broader Nepali population. Second, it applies an intersectionality framework to examine compounded disadvantages. Third, it extended earlier works [11,13] by using concentration curve and intersectionality analysis to analyze multiple level of marginalization.

This study has some limitations. First, this observational and cross-sectional design pecan shows associations but cannot prove causality or temporality. Second, outcome was self-reported, during face-to-face interviews with respondents, which may introduce social desirability bias. In addition, DHS survey records enrollment at an individual-level, whereas NHIP enrollment is a household decision. Sampling weights provided in the survey were applied to ensure representativeness. Results for women and men are shown separately. Although gender was not a sampling stratum the smaller male sample may slightly reduce precision for male-specific estimates. Finally, the quantitative approach may not fully capture lived experiences of intersecting disadvantages. Further qualitative research would be valuable.

## 5. Conclusions

This study applied an intersectional lens to examine how overlapping inequalities affect NHIP enrollment in Nepal. The findings show that social identity does not operate in isolation. Instead, poverty, illiteracy and ethnic marginalization interact to create compounded disadvantages. Individuals facing these compounded disadvantages had substantially lower enrollment than those with no disadvantage. These inequities reflect systemic barriers that disproportionately exclude the most vulnerable populations from financial risk protection. The results highlight that although the NHIP is moving towards UHC, it currently fails to reach those who need it most because of multiple forms of marginalization.

## Figures and Tables

**Table 1:** Bivariate analysis for association between coverage of NHIP and explanatory variables.

	Men			Women		
Variable	N	Coverage % [95% CI]	*p* Value	N	Coverage % [95% CI]	*p* Value
Overall	4913	10.2 [8.8, 11.8]		14,845	10.8 [9.6, 12.2]	
Structural						
Ethnicity						
Disadvantaged	3311	7.5 [6.3, 9.0]		9937	7.3 [6.3, 8.5]	
Advantaged	1602	15.8 [13.0, 19.1]	**<0.001**	4908	17.8 [15.5, 20.5]	**<0.001**
Education						
Illiterate	393	2.5 [1.2, 5.1]		3796	4.5 [3.6, 5.6]	
Literate	4520	10.9 [9.4, 12.6]	**<0.001**	11,049	13.0 [11.5, 14.6]	**<0.001**
Wealth rank						
Poor (40%)	1684	6.8 [5.4, 8.5]		5485	5.8 [4.8, 7.0]	
Rich (60%)	3229	12.0 [10.2, 14.2]	**<0.001**	9360	13.7 [12.1, 15.6]	**<0.001**
Intersectionality						
Poor, illiterate and disadvantaged ethnicity	241	3.0 [1.2, 7.0]		1600	3.4 [2.4, 4.9]	
Poor, illiterate and advantaged ethnicity	21	7.2 [1.7, 25.6]		450	5.8 [4.0, 8.3]	
Poor, literate and disadvantaged ethnicity	1002	6.7 [5.0, 9.0]		2361	5.7 [4.5, 7.3]	
Rich, illiterate and disadvantaged ethnicity	121	0		1503	4.1 [3.1, 5.5]	
Poor, literate and advantaged ethnicity	420	9.3 [6.7, 12.7]	**<0.001**	1074	9.7 [7.6, 12.2]	**<0.001**
Rich, illiterate and advantaged ethnicity	10	13.4 [1.9, 55.6]		243	11.5 [7.3, 17.5]	
Rich, literate and disadvantaged ethnicity	1947	9.0 [7.2, 11.1]		4473	10.7 [9.0, 12.5]	
Rich, literate and advantaged ethnicity	1151	18.4 [14.9, 22.6]		3142	22.9 [19.6, 26.5]	
Marginalization						
Triple disadvantage	241	3.0 [1.2, 7.0]		1600	3.4 [2.4, 4.9]	
Double disadvantage	1145	6.0 [4.5, 8.0]	**<0.001**	4314	5.2 [4.3, 6.3]	**<0.001**
Single disadvantage	2376	9.0 [7.5, 10.9]		5789	10.5 [9.1, 12.1]	
No disadvantage	1151	18.4 [14.9, 22.6]		3142	22.9 [19.6, 26.5]	
Intermediatory						
Province						
Koshi	882	21.8 [17.0, 27.5]		2493	20.4 [16.5, 25.0]	
Madhesh	997	3.1 [1.9, 5.2]		3010	2.7 [1.6, 4.4]	
Bagmati	1214	8.4 [5.9, 11.7]		3062	11.5 [8.5, 15.2]	
Gandaki	387	11.7 [8.3, 16.3]	**<0.001**	1401	16.6 [13.2, 20.7]	**<0.001**
Lumbini	812	9.0 [5.8, 13.7]		2691	9.4 [6.7, 13.0]	
Karnali	266	12.3 [8.4, 17.5]		909	10.3 [7.5, 14.1]	
Sudhurpaschim	355	7.3 [4.1, 12.7]		1279	6.6 [4.0, 10.9]	
Residence						
Urban	3462	11.0 [9.2, 13.1]	0.070	10,178	12.2 [10.5, 14.1]	
Rural	1451	8.4 [6.6, 10.6]		4667	7.8 [6.4, 9.4]	**<0.001**
Ecological zone						
Mountain	255	10.7 [5.8, 18.8]		791	9.9 [6.7, 14.4]	
Hill	1973	10.3 [8.3, 12.6]	0.988	5872	11.7 [9.9, 13.9]	0.416
Terai	2685	10.2 [8.2, 12.5]		8182	10.2 [8.5, 12.2]	

Note: The *p*-value in bold indicates significant values.

**Table 2:** Socioeconomic and education-based Erreygers Normalized Concentration Index (ENCI) in the enrollment of NHIP.

Characteristics	ENCI (SE)			
	Socioeconomic Inequality		Education-Based Inequality	
	Men	Women	Men	Women
Overall	0.071 *** (0.011)	0.102 *** (0.005)	0.101 *** (0.009)	0.105 *** (0.005)
Place of residence				
Urban	0.063 *** (0.014)	0.104 *** (0.008)	0.087 *** (0.013)	0.115 *** (0.008)
Rural	0.060 *** (0.013)	0.077 *** (0.007)	0.111 *** (0.012)	0.083 *** (0.007)
Ecological zone				
Mountain	0.046 (0.033)	0.059 *** (0.016)	0.084 * (0.036)	0.043 * (0.019)
Hill	0.086 *** (0.015)	0.129 *** (0.008)	0.097 *** (0.014)	0.098 *** (0.008)
Terai	0.111 *** (0.013)	0.121 *** (0.008)	0.109 *** (0.013)	0.122 *** (0.007)
Province				
Koshi	0.303 *** (0.030)	0.314 *** (0.018)	0.199 *** (0.029)	0.188 *** (0.017)
Madhesh	0.019 (0.013)	0.026 *** (0.007)	0.051 *** (0.013)	0.033 *** (0.006)
Bagmati	0.080 *** (0.022)	0.117 *** (0.014)	0.128 *** (0.021)	0.102 *** (0.014)
Gandaki	0.045 (0.033)	0.102 *** (0.020)	0.128 *** (0.029)	0.131 *** (0.019)
Lumbini	0.064 * (0.025)	0.073 *** (0.014)	0.063 ** (0.023)	0.092 *** (0.013)
Karnali	0.047 (0.026)	0.057 *** (0.012)	0.066 * (0.028)	0.036 * (0.014)
Sudhurpaschim	0.023 (0.024)	0.046 *** (0.012)	0.018 (0.023)	0.040 *** (0.011)
Marginalization				
Triple disadvantage	0.032 (0.029)	0.001 (0.944)	0.000	0.000
Double disadvantage	−0.015 (0.016)	0.011 (0.007)	0.071 *** (0.015)	0.022 ** (0.007)
Single disadvantage	0.027 * (0.013)	0.053 *** (0.009)	0.076 *** (0.012)	0.067 *** (0.008)
No disadvantage	0.079 ** (0.026)	0.068 *** (0.017)	0.082 *** (0.024)	0.064 *** (0.016)

Note: SE, standard errors; * *p* < 0.05, ** *p* < 0.01, *** *p* < 0.001.

**Table 3:** Unadjusted and adjusted odds ratio of NHIP coverage among women and men.

	Men (N = 4913)		Women (N = 14,845)	
	uOR [95% CI]	aOR [95% CI]	uOR [95% CI]	aOR [95% CI]
Marginalization				
Triple disadvantage	Ref	Ref	Ref	Ref
Double disadvantage	2.1 [0.8, 5.2]	1.6 [0.6, 4.3]	1.9 *** [1.3, 2.7]	1.6 * [1.1, 2.3]
Single disadvantage	3.1 * [1.3, 7.9]	2.9 * [1.1, 7.6]	3.9 *** [2.5, 6.1]	3.3 *** [2.1, 5.3]
No disadvantage	7.0 *** [2.8, 17.6]	6.4 *** [2.4, 17.0]	9.9 *** [6.2, 15.7]	8.0 *** [4.9, 13.2]
Residence				
Urban	Ref	Ref	Ref	Ref
Rural	0.7 [0.5, 1.0]	0.8 [0.5, 1.0]	0.6 *** [0.5, 0.8]	0.7 ** [0.5, 0.9]
Ecological zone				
Mountain	Ref	Ref	Ref	Ref
Hill	1.0 [0.5, 1.0]	0.9 [0.4, 1.9]	1.2 [0.8, 1.9]	0.8 [0.5, 1.4]
Terai	0.9 [0.5, 1.9]	1.0 [0.4, 2.3]	1.0 [0.6, 1.7]	1.0 [0.5, 1.7]
Province				
Koshi	Ref	Ref	Ref	Ref
Madhesh	0.1 *** [0.1, 0.2]	0.1 *** [0.1, 0.2]	0.1 *** [0.1, 0.2]	0.1 *** [0.1, 0.2]
Bagmati	0.3 *** [0.2, 0.5]	0.2 *** [0.1, 0.4]	0.5 ** [0.3, 0.8]	0.3 *** [0.2, 0.5]
Gandaki	0.5 ** [0.3, 0.8]	0.4 ** [0.2, 0.8]	0.8 [0.5, 1.1]	0.7 [0.4, 1.1]
Lumbini	0.4 *** [0.2, 0.6]	0.3 *** [0.2, 0.5]	0.4 *** [0.3, 0.6]	0.4 *** [0.2, 0.5]
Karnali	0.5 ** [0.3, 0.8]	0.5 * [0.3, 0.9]	0.4 *** [0.3, 0.7]	0.5 * [0.3, 0.9]
Sudhurpaschim	0.3 *** [0.2, 0.4]	0.2 *** [0.1, 0.5]	0.3 *** [0.2, 0.5]	0.3 *** [0.1, 0.5]

*** *p* < 0.001, ** *p* < 0.01, * *p* < 0.05.

## Data Availability

Upon approval of our request to access the data, we downloaded NDHS 2022 dataset from https://dhsprogram.com/data/available-datasets.cfm. Data was accessed on 24 June 2023.

## Supplementary Materials

The following supporting information can be downloaded at https://www.mdpi.com/article/10.3390/ijerph23040521/s1. Figure S1: Flowchart showing the sampling of study; Table S1: Description and categorization of covariates; Table S2: Bivariate analysis for association between coverage of NHIP and explanatory variables; Table S3: Unadjusted and Adjusted odds ratio of NHIP coverage among women and men; Table S4: District level service data; Table S5: Province level service data; Analysis S1: Analysis of health insurance service data. Figure S2: Enrolment in National Health Insurance Program by districts and provinces. Figure S3: Enrolment percentage and health facility density by districts and province levels.

## Abbreviations

The following abbreviations are used in this manuscript:

aOR	Adjusted Odds Ratio
CI	Confidence Interval
DHS	Demographic and Health Survey
EA	Enrollment Assistant
ENCI	Erreygers Normalized Concentration Index
FCHV	Female Community Health Volunteer
FSP	First Service Point
HDI	Human Development Index
HIB	Health Insurance Board
LMIC	Low- and Middle-Income Country
MICS	Multiple Indicator Cluster Survey
NDHS	Nepal Demographic and Health Survey
NHIP	National Health Insurance Program
OOPE	Out-of-Pocket Expenditure
PHC	Primary Healthcare Center
SDG	Sustainable Development Goal
SE	Standard Error
UHC	Universal Health Coverage
uOR	Unadjusted Odds Ratio
VIF	Variance Inflation Factor
WHO	World Health Organization
