# Supplementary material for: Inequalities in Enrollment in Nepal’s National Health Insurance Program: An Intersectional Analysis of Nepal Demographic and Health Survey 2022
Source: Int J Environ Res Public Health. 2026 Apr 17;23(4):521. doi: 10.3390/ijerph23040521 (PMC13115810; doi:10.3390/ijerph23040521)
Supplement: Supplementary file 1 [file ijerph-23-00521-s001.zip › ijerph-4131944-supplementary.pdf]

Supplementary File

Inequalities in enrollment in Nepal's national health insurance program: An intersectional analysis of Nepal Demographic and Health Survey 2022

Authors and affiliation

Geha Nath Khanal[1*], Kiran Acharya[2]

[1] School of Nursing, Midwifery, Allied and Public Health, Canterbury Christ Church University, Canterbury, United Kingdom

[2] New ERA, Rudramati Marga, Kalopul, Kathmandu, Nepal

**Corresponding author*

Canterbury Christ Church University, Canterbury, United Kingdom

Email: khanalg@outlook.com

Supplementary Figure

Figure S1: Flowchart showing the sampling of study

Supplementary Tables

Table S1: Description and categorization of covariates

Table S2: Bivariate analysis for association between coverage of NHIP and explanatory variables

Table S3: Unadjusted and Adjusted odds ratio of NHIP coverage among women and men

Table S4: District level service data

Table S5: Province level service data

Supplementary Analysis

Analysis S1: Analysis of health insurance service data

Figure S2: Enrolment in National Health Insurance Program by districts and provinces

Figure S3: Enrolment percentage and health facility density by districts and province levels

Supplementary Figure

Figure S1: Flowchart showing the sampling of study

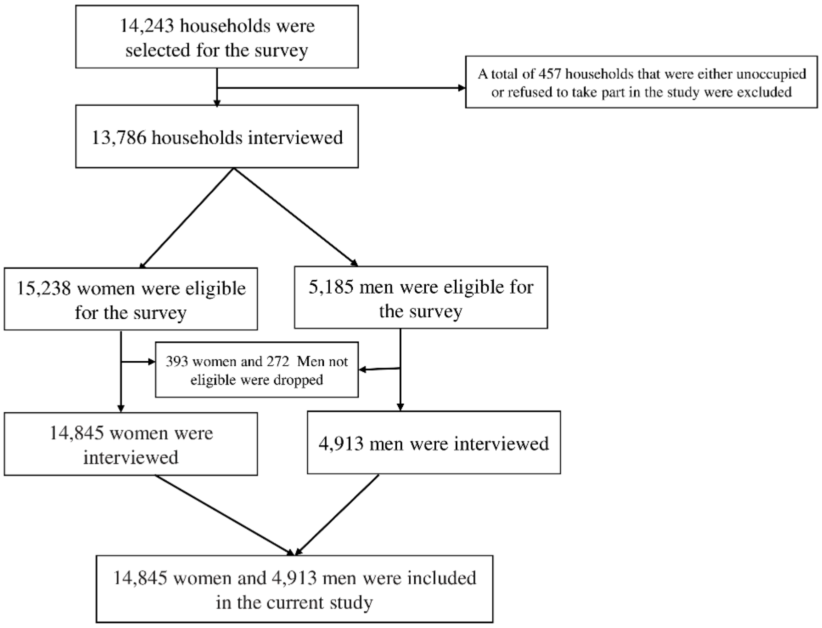

Table S1: Description and categorization of covariates

Variable	Description	Categorization
**Age**<br>**(women and men)**	This variable was defined as the age of women and men at the time of the survey, measured in completed years.	1. 15-19<br>2. 20-24<br>3. 25-29<br>4. 30-34<br>5. 35-39<br>6. 40-44<br>7. 45-49
**Marital status**<br>**(women and men)**	This variable refers to the marital status of women and men at the time of the survey. The 'Formally Married' category includes individuals who are currently married, widowed, divorced, or separated.	1. Never married<br>2. Currently Married<br>3. Formally married
**Household members**<br>**(women and men)**	All members who stayed in the selected households the night before the interview.	1. 1-2 members<br>2. 3-5 members<br>3. More than 5 members
**Female Household head**<br><br>**(women and men)**	This categorization is based on the response to the household interview, regardless of whether it was completed by a woman or a man. It reflects whether the head of the household is female or not.	1. No<br>2. Yes
**Education status**<br><br>**(women and men)**	This variable is based on the revised education system in Nepal, following the Eighth Amendment to the Education Act in 2016 (Ministry of Education, Science and Technology, 2021). As a result, a new classification system was introduced: No Education, Basic (Grades 1–8), Secondary (Grades 9–12), and More than Secondary (Grade 13 and above). The 'No Education' category includes individuals who have never attended school as well as those who have only attended informal preschool programs.	1. No Education<br>2. Basic education<br>3. Secondary education<br>4. Higher
**Education**<br><br>**(women and men)**<br><br>**Dichotomous variable) ***	Education was dichotomized into illiterate (unable to read and write) and literate (able to read and write and having completed at least primary education) in line with NDHS definitions	1. Illiterate<br>2. Literate
**Occupation**<br>**(women and men)**	Women and men aged 15–49 who were currently employed or had worked in the 12 months preceding the survey were asked about their type of occupation. The response options included: professional/technical/managerial, clerical, sales	1. Agriculture<br>2. Not working<br>3. Professional/Clerical/ Sales/Service

Variable	Description	Categorization
	and services, skilled manual, unskilled manual, agriculture, and other.	4. Skilled/Unskilled manual workers
**Ethnicity (women and men)**	Nepal is a multi-ethnic country with diverse languages, religions, and cultural traditions, boasting more than 100 distinct ethnic or caste groups.	1. Dalits<br>2. Madheshi<br>3. Janajati<br>4. Advance Groups<br>5. Muslim<br>6. Newar<br>7. Others
**Ethnicity***<br><br>**(women and men)**<br><br>**Dichotomous variable)**	The Government of Nepal classifies ethnic groups into six broad categories: Dalits, disadvantaged Janajatis (indigenous groups), disadvantaged non-Dalit Terai caste groups, religious minorities (Muslims), relatively advantaged Janajatis, and Brahmins/Chhetris. For this study, these six categories were merged into two groups: disadvantaged ethnicities (Dalits, Muslims, Terai caste groups, and disadvantaged Janajatis) and advantaged ethnicities (Brahmins/Chhetris and relatively advantaged Janajatis).	1. Disadvantaged<br>2. Advantaged
**Religion (women and men)**	According to the Constitution of Nepal 2015, Nepal is a secular state, whereby "secular" denotes the protection of religious and cultural freedoms, including those passed down through generations.	1. Hindu<br>2. Other than Hindu
**Residence (women and men)**	Administrative divisions in Nepal were classified into urban and rural categories. Metropolitan cities, submetropolitan cities, and municipalities were considered urban, and rural municipalities were deemed rural. According to the 2021 census, out of the 6,666,937 households in Nepal, 4,479,662 resided in urban municipalities and 2,187,275 in rural municipalities.	1. Urban<br>3. Rural
**Ecological zone (women and men)**	Nepal, known as a Himalayan country, boasts a remarkably diverse geography. It is divided into three main ecoregions (i.e., ecological zones), rather than administrative divisions. Mountain refers to the massive glacier-encrusted Himalayan region in the north. Hill is the Himalayan foothills, also referred to as the Middle Hills. Terai (or Tarai), a belt of jungle and wetlands harboring tigers and elephants, is situated in the flat Ganges Plain in the south.	1. Mountain<br>2. Hill<br>2. Terai
**Province (women and men)**	In September 2015, Nepal's Constitution introduced the current system of seven provinces, replacing an	1. Koshi<br>2. Madhesh

Variable	Description	Categorization
	earlier arrangement that divided the country into 14 administrative zones and five development regions.	3. Bagmati<br>4. Gandaki<br>5. Lumbini<br>6. Karnali<br>7. Sudurpaschim
**Media exposure (women and men)**	This variable was constructed based on the frequency of respondents 'exposure to radio, television, and newspaper.	3. Not at all<br>4. Less than once a week<br>5. At least once a week
**Internet exposure (women and men)**	This variable represented the frequency of internet use in the month preceding the survey.	1. Not at all<br>2. Less than once a week<br>3. At least once a week<br>4. Almost everyday
**Wealth quintile (women and men)**	The wealth index was calculated using easy-to-collect data on a household's ownership of selected assets, such as televisions and bicycles, materials used for housing construction, water access, and sanitation facilities. With these proxy indicators, households were classified into five quintiles using the standard DHS wealth index. Household wealth index scores were derived using principal component analysis. National wealth quintiles were compiled by assigning the household score to each usual (de jure) household member, ranking each person in the household population by their score, and then dividing the distribution into five equal quintiles (each comprising 20% of the population).	1. Lowest<br>2. Second<br>3. Middle<br>4. Fourth<br>5. Highest
**Wealth* (dichotomous variable)**	The five wealth quintiles defined in the 2022 NDHS were dichotomized into two groups by merging the lowest two quintiles (lower 40%) as "lower wealth status" and the highest three quintiles (upper 60%) as "upper wealth status".	1. Poor (40%)<br>**2.** Rich (60%)
**Self-reported health status (women and men)**	Women and men aged 15–49 who were currently employed or had worked in the 12 months preceding the survey were asked about their type of occupation. The response options included: professional/technical/managerial, clerical, sales and services, skilled manual, unskilled manual, agriculture, and other.	1. Very good<br>2. Good<br>3. Moderate<br>4. Bad/very bad
**Functional disability or impairment (women and men)**<br>*Due to missing cases in the women's dataset, which led to a reduced*	The 2022 NDHS included The DHS Program's Disability Module, which comprises a series of questions based on the Washington Group on Disability Statistics Short Set on Functioning (WG-SS). These questions follow the framework of the World Health Organization's International	1. No difficulty<br>2. Have difficulty

Variable	Description	Categorization
sample size, this variable is excluded from the multivariable analysis.	Classification of Functioning, Disability, and Health. The module addresses six core functional domains—seeing, hearing, communication, cognition, walking, and self-care—and provides essential information on disability status. In this study, we categorized individuals as having a disability if they were reported to have difficulty in more than one domain. Further details are available in the final 2022 NDHS report, which is published online.	
**Tobacco consumption (women and men)**	This variable includes current use of both smoked and smokeless tobacco by women and men. Smokeless tobacco use includes snuff by mouth, snuff by nose, chewing tobacco (such as guthka or khaini), and betel quid with tobacco. Smoked tobacco includes all forms of smokeless tobacco in addition to cigarettes, pipes, cigars/bidis, and water pipes.	1. No<br>2. Yes
**Alcohol consumption (women and men)**	This binary variable indicated whether respondents consumed alcohol in the last month.	1. No<br>2. Yes
**Have symptoms of anxiety or depression (women and men)**	The 2022 NDHS used the Generalized Anxiety Disorder 7-item scale (GAD-7) to assess symptoms of anxiety, and the Patient Health Questionnaire-9 (PHQ-9) to assess symptoms of depression. Based on these tools, respondents with a GAD-7 score of 6 or higher were categorized as having symptoms of anxiety, while those with a PHQ-9 score of 10 or higher were categorized as having symptoms of depression. Further details on the GAD-7 and PHQ-9 are available in the final 2022 NDHS report, which is published online.	1. No<br>2. Yes
**Injured in a road traffic accident in the last 12 months (women and men)**	This variable includes women and men who reported being injured in a road traffic accident within the last 12 months. Injuries included cuts or open wounds, broken bones, burns, head injuries, internal injuries, and similar types of harm.	1. No<br>2. Yes

Variable	Description	Categorization
**Hypertension**<br><br>**(Only women)**<br><br>*In the 2022 NDHS, blood pressure measurements for hypertension were conducted exclusively in a subsample that did not overlap with the men's survey sample. Conversely, health insurance-related questions were included in the subsample selected for the men's survey.*<br><br>*Since this variable is only available in the women's dataset and the sample size is low, it is excluded from the multivariable model.*	Women and men were classified as having hypertension if, at the time of the survey, they had an average systolic blood pressure (SBP) of 140 mmHg or higher, an average diastolic blood pressure (DBP) of 90 mmHg or higher, or if they had normal blood pressure but were currently taking antihypertensive medication. Three blood pressure measurements were taken from each eligible respondent, and the average of the second and third systolic and diastolic readings was used for classification. It is important to note that the term 'hypertension' in this report does not represent a clinical diagnosis, but rather a statistical description of the survey population at the time of data collection. Valid blood pressure measurements were obtained during the survey, and detailed procedures can be found in the final 2022 NDHS report published online.	1. No<br>2. Yes
**Distance to health facility**<br><br>**(Only women)**<br><br>*This question was asked only to women as part of the maternal and newborn health section.*<br><br>*Since this variable is only available in the women's dataset and the sample size is low, it was excluded from the multivariable model.*	This question was asked only to women: *'How long does it take, in minutes, to travel from your home to the nearest healthcare facility, which could be a hospital, a primary health care center, a medical doctor, or a health post?'* Additionally, respondents were asked about the type of facility closest to them. Based on their responses, we categorized the nearest healthcare facilities as Primary Health Care Centers (PHCCs) and above, which are the public facilities where the national health insurance program is implemented. Travel time to these facilities was then grouped into three categories based on the number of minutes taken to reach them.	1. Within 30 minutes<br>2. 30-60 minutes<br>3. Above 60 minutes
**Intersectionality^ variables (women and men)**	Three dichotomous variables above (wealth, ethnicity and education) were combined to construct eight different marginalization status variables.	1. **Poor, illiterate and disadvantaged** ethnicity<br>2. **Poor, illiterate** and advantaged ethnicity

Variable	Description	Categorization
		3. **Poor,** literate and **disadvantaged ethnicity**<br>4. Rich, **illiterate and disadvantaged ethnicity**<br>5. **Poor,** literate and advantaged ethnicity<br>6. Rich, **illiterate** and advantaged ethnicity<br>7. Rich, literate and **disadvantaged ethnicity**<br>8. Rich, literate and advantaged ethnicity
**Marginalization variables (women and men)**	The eight original categories of marginalization status variables above were subsequently collapsed into four categories, based on the number of concurrent disadvantages a woman experienced: triple, double, single, or no disadvantages	1. Triple disadvantage<br>2. Double disadvantage<br>3. Single disadvantage<br>4. No disadvantage

^ *number of bold variables in categorization indicates number of disadvantages*

Table S2: Bivariate analysis for association between coverage of NHIP and explanatory variables

	**Men**			**Women**		
**Variable**	**N**	**Coverage % [95% CI]**	**P value**	**N**	**Coverage% [95% CI]**	**P value**
**Overall**	**4,913**	**10.2 [8.8,11.8]**		**14,845**	**10.8 [9.6,12.2]**	
Age						
15-19	985	6.6 [5.1, 8.6]		2,643	8.0 [6.5,9.9]	
20-24	857	11.1[8.6, 14.2]		2,637	9.5 [8.1,11.3]	
25-29	716	8.8 [6.6, 11.7]		2,435	11.9 [10.0,14.0]	
30-34	616	9.9 [7.4, 13.1]		2,144	11.9 [10.1,14.1]	
35-39	639	14.2[11.4, 17.5]	**<0.001**	2,025	11.1 [9.3,13.2]	**<0.001**
40-44	604	11.0 [8.4, 14.3]		1,629	12.5 [10.3,15.1]	
45-49	496	12.3 [8.8, 11.8]		1,332	12.6 [10.5,15.1]	
Marital Status						
Never married	1,768	9.0 [7.3,11.0]		3,203	10.9 [9.1,13.0]	
Currently married	3,101	11.0 [9.4,12.7]	0.091	11,180	10.8 [9.6,12.2]	0.936
Formally married	44	8.3 [2.9,21.3]		462	10.3 [7.5,13.9]	
Household Members						
1-2 members	377	11.3 [8.0,15.7]		1,326	9.4 [7.5,11.7]	
3-5 members	2,901	10.3 [8.6,12.3]	0.830	8,562	11.9 [10.4,13.7]	**0.002**
More than 5 members	1,635	9.9 [7.7,12.6]		4,957	9.2 [7.8,10.9]	
Female Household head						
No	4,003	10.6 [9.1,12.3]	0.278	9,745	11.3 [9.9,12.9]	0.085
Yes	910	8.7 [6.1,12.2]		5,100	9.8 [8.3,11.5]	
Education status						
No Education	393	2.5 [1.2,5.1]		3,796	4.5 [3.6,5.6]	
Basic education	1,898	6.4 [5.1,8.1]		4,595	7.5 [6.3,8.8]	
Secondary education	2,444	13 [11.0,15.4]	**<0.001**	5,798	16.0 [14.0,18.2]	**<0.001**
Higher	377	20.9 [16.2,26.6]		656	25.1 [20.3,30.5]	
Occupation						
Agriculture	1,161	9.8 [7.7,12.5]		7,146	8.5 [7.3,9.8]	
Not working	672	11.1 [8.1,14.9]		4,147	9.3 [7.6,11.3]	

	Men			Women		
**Variable**	**N**	**Coverage % [95% CI]**	**P value**	**N**	**Coverage% [95% CI]**	**P value**
Professional/Clerical/ Sales/Service	1,268	15.3 [12.7,18.4]	**<0.001**	2,317	21.7 [18.6,25.1]	**<0.001**
Skilled/Unskilled manual workers	1,812	6.6 [5.2,8.5]		1,235	8.9 [6.5,12.1]	
Ethnicity						
Dalits	658	10 [7.3,13.5]	**<0.001**	2,240	7.6 [6.0,9.5]	**<0.001**
Madheshi	799	4.4 [2.8,6.9]		2,152	5.2 [3.7,7.3]	
Janajati	1,626	8.4 [6.6,10.6]		4,869	8.8 [7.2,10.7]	
Advance Groups	1,351	16.4 [13.5,19.9]		4,333	17.7 [15.3,20.4]	
Muslim	228	5.3 [2.3,12.1]		676	2.6 [1.3,5.3]	
Newar	244	12.5 [6.4,22.9]		559	18.3 [11.1,28.7]	
Others	8	18.4 [2.2,69.0]		15	41.7 [13.7,76.3]	
Religion						
Hindu	4,025	10.8 [9.3,12.5]	**0.040**	12,374	11.6 [10.2,13.0]	**<0.001**
Other than Hindu	888	7.5 [5.2,10.6]		2,471	7.1 [5.5,9.2]	
Residence						
Urban	3,462	11.0 [9.2,13.1]	0.070	10,178	12.2 [10.5,14.1]	**<0.001**
Rural	1,451	8.4 [6.6,10.6]		4,667	7.8 [6.4,9.4]	
Ecological zone						
Mountain	255	10.7 [5.8,18.8]	0.988	791	9.9 [6.7,14.4]	0.416
Hill	1,973	10.3 [8.3,12.6]		5,872	11.7 [9.9,13.9]	
Terai	2,685	10.2 [8.2,12.5]		8,182	10.2 [8.5,12.2]	
Province						
Koshi	882	21.8 [17.0,27.5]	**<0.001**	2,493	20.4 [16.5,25.0]	**<0.001**
Madhesh	997	3.1 [1.9,5.2]		3,010	2.7 [1.6,4.4]	
Bagmati	1,214	8.4 [5.9,11.7]		3,062	11.5 [8.5,15.2]	
Gandaki	387	11.7 [8.3,16.3]		1,401	16.6 [13.2,20.7]	
Lumbini	812	9.0 [5.8,13.7]		2,691	9.4 [6.7,13.0]	
Karnali	266	12.3 [8.4,17.5]		909	10.3 [7.5,14.1]	
Sudhurpaschim	355	7.3 [4.1,12.7]		1,279	6.6 [4.0,10.9]	
Media exposure						
Not at all	747	6.4 [3.9,10.3]		3,135	6.3 [5.1,7.8]	
Less than once a week	2,563	10.1 [8.4,12.2]	**0.021**	6,957	11.9 [10.4,13.5]	**<0.001**

	Men			Women		
**Variable**	**N**	**Coverage % [95% CI]**	**P value**	**N**	**Coverage% [95% CI]**	**P value**
At least once a week	1,603	12.2 [10.0,14.7]		4,753	12.2 [10.5,14.1]	
Internet exposure						
Not at all	1,319	5.5 [4.2,7.2]		5,672	6.5 [5.5,7.6]	
Less than once a week	178	7.8 [4.4,13.4]		854	7.1 [5.3,9.3]	
At least once a week	805	10.2 [8.1,12.9]	**<0.001**	2,161	9.3 [7.8,11.1]	**<0.001**
Almost everyday	2,611	12.8 [10.8,15.0]		6,158	15.8 [13.7,18.2]	
Wealth Quintile						
Lowest	751	5.1 [3.7,7.0]		2,628	4.1 [3.3,5.2]	
Second	933	8.2 [6.2,10.8]		2,857	7.4 [5.9,9.3]	
Middle	957	8.2 [6.4,10.5]		3,028	8.0 [6.6,9.7]	
Fourth	1,135	10.8 [8.3,13.8]	**<0.001**	3,197	13.1 [11.0,15.4]	**<0.001**
Highest	1,137	16.5 [13.0,20.7]		3,135	20.0 [16.5,24.0]	
Self-reported health status						
Very good	423	7.5 [4.9,11.3]		823	5.9 [4.0,8.5]	
Good	1,913	10.8 [8.9,13.1]		4,168	8.9 [7.4,10.5]	
Moderate	2,348	10.0 [8.3,12.1]		8,423	11.9 [10.5,13.6]	
Bad/very bad	230	12.3 [8.2,18.1]	0.315	1,429	12.7 [10.6,15.1]	**<0.001**
Functional disability or impairment						
No difficulty	4,094	10.0 [8.6,11.7]		5,828	11.1 [9.7,12.7]	
Have difficulty	817	11.1 [8.8,14.0]	0.357	1,581	10.8 [8.9,13.0]	0.742
Tobacco consumption						
No	2,477	12.4 [10.6,14.4]	**<0.001**	13,744	11.0 [9.8,12.5]	**0.014**
Yes	2,436	8.1 [6.5,9.9]		1,101	8.0 [6.0,10.4]	
Alcohol consumption in last month						
No	2,836	11.3 [9.6,13.4]	**0.026**	13,226	11.0 [9.8,12.4]	**0.025**
Yes	2,077	8.7 [7.0,10.7]		1,619	9.0 [7.2,11.1]	
Have symptoms of anxiety or depression						
No	4,347	9.9 [8.5,11.4]	0.059	13,192	10.8 [9.5,12.2]	0.711
Yes	566	13.1 [9.7,17.5]		1,653	11.2 [8.9,14.0]	
Injured in a road traffic accident in the last 12 months						

	Men			Women		
**Variable**	**N**	**Coverage % [95% CI]**	**P value**	**N**	**Coverage% [95% CI]**	**P value**
No	4,409	10.1 [8.7,11.8]	0.607	13,260	10.6 [9.4,11.9]	0.104
Yes	504	11.1 [8.0,15.1]		1,585	12.7 [10.0,16.1]	
Hypertension^						
No	NA	NA	NA	3,621	11.2 [9.4,13.3]	
Yes				364	12.3 [8.6,17.3]	0.622
Distance to health facility ^^						
With in 30 minutes				1,285	15.1 [11.6,19.4]	
30-60 minutes	NA	NA	NA	51	2.4 [0.8,7.0]	**0.002**
Above 60 minutes				27	5.7 [1.4,19.7]	

NA=Not Applicable

Bold p-values indicate statistical significance at p < 0.05

^ Hypertension status was assessed for 3,986 women,

^^ The distance to the nearest health facility—a Primary Healthcare Center (PHCC) or higher-level facility—was assessed for 1,364 women who reported it as their closest healthcare option.

Table S3: Unadjusted and Adjusted odds ratio of NHIP coverage among women and men

	Men (N=4913)		Women (N=14,845)	
	**UOR [95% CI]**	**AOR [95% CI]**	**UOR [95% CI]**	**AOR [95% CI]**
Age				
15-19	Ref	Ref	Ref	Ref
20-24	1.8**[1.2-2.5]	2.0***[1.4 - 3.0]	1.2[1.0-1.5]	1.0[0.8 - 1.4]
25-29	1.4 [0.9-2.0]	1.5[0.9 - 2.6]	1.5*** [1.2-2.0]	1.3[1.0 - 1.9]
30-34	1.6* [1.1-2.3]	1.6[1.0 - 2.7]	1.6** [1.2-2.0]	1.4[1.0 - 2.0]
35-39	2.3***[1.7-3.3]	3.0***[1.9 - 4.9]	1.4***[1.2-1.7]	1.5*[1.1 - 2.1]
40-44	1.7** [1.2-2.5]	2.2**[1.3 - 3.7]	1.6*** [1.3-2.1]	2.1***[1.4 - 3.1]
45-49	2.0*** [1.3-2.9]	2.4**[1.3 - 4.4]	1.7*** [1.3-2.1]	2.2***[1.5 - 3.4]
Marital Status				
Never married	Ref	Ref	Ref	Ref
Currently married	1.2* [1.0-1.5]	1.4[1.0 - 1.8]	1.0 [0.8-1.2]	1.0[0.8 - 1.2]
Formally married	0.9 [0.3-2.7]	1.2[0.3 - 4.5]	0.9 [0.7-1.3]	1.0[0.6 - 1.5]
Household Members				
1-2 members	Ref	Ref	Ref	Ref
3-5 members	0.9 [0.6-1.4]	0.9[0.6 - 1.4]	1.3* [1.0-1.7]	1.4*[1.1 - 1.8]
More than 5 members	0.9 [0.5-1.4]	1.0[0.6 - 1.7]	1.0 [0.7-1.3]	1.3[1.0 - 1.8]
Female Household head				
No	Ref	Ref	Ref	Ref
Yes	0.8 [0.5-1.2]	0.8 [0.6-1.2]	0.9 [0.7-1.0]	0.9[0.7 - 1.1]
Education status				
No Education	Ref	Ref	Ref	Ref
Basic education	2.6** [1.3-5.4]	2.0[0.9 - 4.4]	1.7*** [1.3-2.2]	1.4*[1.0 - 1.8]
Secondary education	5.7***[2.8-12.0]	3.1**[1.3 - 7.2]	4.1***[3.2-5.1]	2.6***[1.9 - 3.5]
Higher	10.2***[4.7-22.2]	4.0**[1.6 - 9.6]	7.1***[5.1-9.9]	3.0***[2.1 - 4.3]
Occupation				
Agriculture	Ref	Ref	Ref	Ref
Not working	1.1[0.8-1.7]	1.6[1.0 - 2.6]	1.1[0.9-1.4]	0.7**[0.6 - 0.9]
Professional/Clerical/ Sales/Service	1.7***[1.2-2.2]	1.1[0.7 - 1.5]	3.0***[2.4-3.7]	1.4**[1.1 - 1.7]
Skilled/Unskilled manual workers	0.7*[0.5-0.9]	0.8[0.5 - 1.1]	1.1[0.7-1.5]	1.0[0.7 - 1.4]
Ethnicity				

	Men (N=4913)		Women (N=14,845)	
	**UOR [95% CI]**	**AOR [95% CI]**	**UOR [95% CI]**	**AOR [95% CI]**
Dalits	Ref	Ref	Ref	Ref
Madheshi	0.4**[0.2-0.7]	0.4**[0.2 - 0.7]	0.7[0.4-1.0]	0.7[0.5 - 1.1]
Janajati	0.8[0.6-1.2]	0.4***[0.3 - 0.6]	1.2[0.9-1.6]	0.7*[0.5 - 0.9]
Advance Groups	1.8**[1.2-2.6]	0.7*[0.4 - 1.0]	2.6***[2.0-3.5]	1.3[0.9 - 1.7]
Muslim	0.5[0.2-1.3]	0.4[0.2 - 1.2]	0.3**[0.1-0.7]	0.5[0.2 - 1.1]
Newar	1.3[0.6-2.9]	0.6[0.3 - 1.4]	2.7**[1.4-5.2]	1.1[0.6 - 2.2]
Others	2.0[0.2-12.2]	1.3[0.1 - 12.5]	8.7**[2.0-39.0]	4.1[0.7 - 23.2]
Religion				
Hindu	Ref	Ref	Ref	Ref
Others	0.7* [0.4-1.0]	0.8 [0.4-1.4]	0.6*** [0.4-0.8]	0.8[0.6 - 1.1]
Residence				
Urban	Ref	Ref	Ref	Ref
Rural	0.7[0.5-1.0]	1.1[0.8-1.5]	0.6*** [0.5-0.8]	1.0[0.8 - 1.3]
Ecological zone				
Mountain	Ref	Ref	Ref	Ref
Hill	1.0[0.5-1.9]	0.8[0.3-1.7]	1.2[0.8-1.9]	0.7[0.4 - 1.2]
Terai	0.9[0.5-1.9]	0.8[0.3-2.2]	1.0[0.6-1.7]	0.8[0.4 - 1.6]
Province				
Koshi	Ref	Ref	Ref	Ref
Madhesh	0.1***[0.1-0.2]	0.1***[0.1 - 0.2]	0.1***[0.1-0.2]	0.1***[0.1 - 0.2]
Bagmati	0.3***[0.2-0.5]	0.2***[0.1 - 0.4]	0.5**[0.3-0.8]	0.3***[0.2 - 0.5]
Gandaki	0.5**[0.3-0.8]	0.3***[0.1 - 0.6]	0.8[0.5-1.1]	0.6*[0.4 - 0.9]
Lumbini	0.4***[0.2-0.6]	0.2***[0.1 - 0.4]	0.4***[0.3-0.6]	0.3***[0.2 - 0.5]
Karnali	0.5**[0.3-0.8]	0.4**[0.2 - 0.8]	0.4***[0.3-0.7]	0.5*[0.3 - 0.9]
Sudhurpaschim	0.3***[0.1-0.6]	0.2***[0.1 - 0.5]	0.3***[0.2-0.5]	0.3***[0.1 - 0.5]
Media exposure				
Not at all	Ref	Ref	Ref	Ref
Less than once a week	2.0*[1.2-3.5]	0.9[0.5 - 1.5]	2.1***[1.6-2.6]	1.2[0.9 - 1.4]
At least once a week	1.6[1.0-2.8]	1.0[0.6 - 1.8]	2.0***[1.6-2.5]	1.1[0.9 - 1.4]
Internet exposure				
Not at all	Ref	Ref	Ref	Ref
Less than once a week	1.4[0.7-2.8]	1.2[0.6-2.3]	1.1[0.8-1.5]	1.0[0.7 - 1.3]

	Men (N=4913)		Women (N=14,845)	
	**UOR [95% CI]**	**AOR [95% CI]**	**UOR [95% CI]**	**AOR [95% CI]**
At least once a week	1.9***[1.4-2.7]	1.8**[1.3-2.6]	1.5***[1.2-1.8]	1.1[0.9 - 1.4]
Almost everyday	2.5***[1.8-3.4]	1.8**[1.2-2.7]	2.7***[2.2-3.3]	1.4**[1.1 - 1.7]
Wealth Quintile				
Lowest	Ref	Ref	Ref	Ref
Second	1.7*[1.1-2.6]	2.0*[1.2 - 3.3]	1.8***[1.4-2.5]	2.1***[1.4 - 2.9]
Middle	1.7*[1.1-2.6]	1.9*[1.1 - 3.5]	2.0***[1.5-2.7]	2.1***[1.4 - 3.1]
Fourth	2.2***[1.5-3.5]	2.0*[1.1 - 3.6]	3.5***[2.6-4.7]	3.0***[2.0 - 4.5]
Highest	3.7***[2.4-5.7]	2.9**[1.5 - 5.4]	5.8***[4.1-8.1]	3.6***[2.4 - 5.4]
Self-reported health status				
Very good	Ref	Ref	Ref	Ref
Good	1.5[0.9-2.5]	1.4[0.8-2.3]	1.6*[1.0-2.4]	1.0[0.6 - 1.5]
Moderate	1.4[0.9-2.2]	1.5[0.9-2.5]	2.2***[1.4-3.2]	1.0[0.7 - 1.6]
Bad/very bad	1.7[0.9-3.3]	1.7=8[0.9-3.7]	2.3***[1.5-3.6]	1.6[1.0 - 2.5]
Tobacco consumption				
No	Ref	Ref	Ref	Ref
Yes	0.6***[0.5-0.8]	0.7**[0.5-0.9]	0.7*[0.5-0.9]	0.9[0.7 - 1.2]
Alcohol consumption in last month				
No	Ref	Ref	Ref	Ref
Yes	1.4[1.0-1.9]	0.9[0.7-1.1]	0.8*[0.6-1.0]	0.8[0.7 - 1.0]
Have symptoms of anxiety or depression				
No	Ref	Ref	Ref	Ref
Yes	1.4[1.0-1.9]	1.3[0.9-1.9]	1.0[0.8-1.3]	1.2[0.9 - 1.5]
Injured in a road traffic accident in the last 12 months				
No	Ref	Ref	Ref	Ref
Yes	1.1[0.8-1.6]	1.2[0.8-1.8]	1.2[1.0-1.6]	1.3[1.0 - 1.7]

*** p<0.001, ** p<0.01, * p<0.05

Table S4: District level service data

Province	District	No of empanneled health facility	Population 2021	Insured Population	% enrolled	Health facility/ 100000 population
Sushurpaschim	Achham	4	228852	60330	26.4	1.7
Lumbini	Arghakhanchi	4	177086	89789	50.7	2.3
Gandaki	Baglung	4	249211	134292	53.9	1.6
Sushurpaschim	Baitadi	3	242157	28917	11.9	1.2
Sushurpaschim	Bajhang	3	189085	45634	24.1	1.6
Sushurpaschim	Bajura	2	138523	38940	28.1	1.4
Lumbini	Banke	8	603194	88557	14.7	1.3
Madhesh	Bara	6	763137	87052	11.4	0.8
Lumbini	Bardiya	5	459900	144313	31.4	1.1
Bagmati	Bhaktapur	12	432132	281134	65.1	2.8
Koshi	Bhojpur	4	157923	44719	28.3	2.5
Bagmati	Chitwan	13	719859	568781	79.0	1.8
Sushurpaschim	Dadeldhura	2	139602	27333	19.6	1.4
Karnali	Dailekh	5	252313	39272	15.6	2.0
Lumbini	Dang	7	674993	70366	10.4	1.0
Sushurpaschim	Darchula	2	133310	19129	14.3	1.5
Bagmati	Dhading	5	325710	91447	28.1	1.5
Koshi	Dhankuta	3	150599	52146	34.6	2.0
Madhesh	Dhanusa	9	867747	68805	7.9	1.0
Bagmati	Dolakha	5	172767	48897	28.3	2.9
Karnali	Dolpa	1	42774	5045	11.8	2.3
Sushurpaschim	Doti	3	204831	23885	11.7	1.5
Gandaki	Gorkha	7	251027	128694	51.3	2.8
Lumbini	Gulmi	6	246494	117467	47.7	2.4
Karnali	Humla	1	55394	16521	29.8	1.8
Koshi	Ilam	8	279534	172715	61.8	2.9
Karnali	Jajarkot	5	189360	54184	28.6	2.6
Koshi	Jhapa	26	998054	766937	76.8	2.6
Karnali	Jumla	2	118349	94536	79.9	1.7
Sushurpaschim	Kailali	11	904666	311041	34.4	1.2
Karnali	Kalikot	3	145292	55701	38.3	2.1
Sushurpaschim	Kanchanpur	4	513757	70016	13.6	0.8
Lumbini	Kapilbastu	6	682961	132585	19.4	0.9
Gandaki	Kaski	15	600051	315296	52.5	2.5
Bagmati	Kathmandu	31	2041587	257814	12.6	1.5
Bagmati	Kavrepalanchok	7	364039	193579	53.2	1.9
Koshi	Khotang	5	175298	65739	37.5	2.9
Bagmati	Lalitpur	11	551667	102024	18.5	2.0
Gandaki	Lamjung	4	155852	56203	36.1	2.6
Madhesh	Mahottari	7	706994	61568	8.7	1.0
Bagmati	Makawanpur	8	466073	242207	52.0	1.7
Gandaki	Manang	1	5658	686	12.1	17.7

Province	District	No of empanneled health facility	Population 2021	Insured Population	% enrolled	Health facility/ 100000 population
Koshi	Morang	25	1148156	606090	52.8	2.2
Karnali	Mugu	2	64549	12205	18.9	3.1
Gandaki	Mustang	2	14452	2121	14.7	13.8
Gandaki	Myagdi	2	107033	39946	37.3	1.9
Lumbini	Nawalparasi (Bardaghat Susta Pashchim)	4	386868	60347	15.6	1.0
Gandaki	Nawalparasi (Bardaghat Susta Purb)	7	378079	165479	43.8	1.9
Bagmati	Nuwakot	4	263391	83011	31.5	1.5
Koshi	Okhaldhunga	3	139552	52829	37.9	2.1
Lumbini	Palpa	7	245027	243179	99.2	2.9
Koshi	Panchthar	3	172400	40611	23.6	1.7
Gandaki	Parbat	3	130887	50999	39.0	2.3
Madhesh	Parsa	10	654471	107367	16.4	1.5
Lumbini	Pyuthan	3	232019	71540	30.8	1.3
Bagmati	Ramechhap	7	170302	78127	45.9	4.1
Bagmati	Rasuwa	2	46689	17835	38.2	4.3
Madhesh	Rautahat	7	813573	99600	12.2	0.9
Lumbini	Rolpa	3	234793	44726	19.0	1.3
Lumbini	East Rukum	2	56786	11405	20.1	3.5
Karnali	West Rukum	7	166740	86383	51.8	4.2
Lumbini	Rupandehi	11	1121957	211037	18.8	1.0
Karnali	Salyan	4	238515	40435	17.0	1.7
Koshi	Sankhuwasabha	6	158041	55202	34.9	3.8
Madhesh	Saptari	7	706255	115221	16.3	1.0
Madhesh	Sarlahi	6	862470	58648	6.8	0.7
Bagmati	Sindhuli	5	300026	123471	41.2	1.7
Bagmati	Sindhupalchowk	5	262624	71479	27.2	1.9
Madhesh	Siraha	7	739953	134412	18.2	0.9
Koshi	Solukhumbu	5	104851	16314	15.6	4.8
Koshi	Sunsari	11	926962	579581	62.5	1.2
Karnali	Surkhet	6	415126	121773	29.3	1.4
Gandaki	Syangja	9	253024	158575	62.7	3.6
Gandaki	Tanahun	6	321153	152014	47.3	1.9
Koshi	Taplejung	4	120590	16910	14.0	3.3
Koshi	Tehrathum	5	88731	24510	27.6	5.6
Koshi	Udayapur	4	340721	84034	24.7	1.2

* Updated data by 5th May 2025

Table S5: Province level service data

Province	Enrolled Population*	Total Population Census 2021)	% Enrolled	Number of Empanelled Health Facility	Health facility/ 100000 population
Bagmati	2159806	6116866	35.30902917	115	1.88
Gandaki	1204305	2466427	48.8279199	60	2.43
Karnali	526055	1688412	31.15679111	36	2.13
Koshi	2578337	4961412	51.96780675	112	2.26
Lumbini	1285311	5122078	25.09354602	66	1.29
Madhesh	732673	6114600	11.98235371	59	0.96
Sudhurpaschim	625225	2694783	23.20131157	34	1.26

* Updated data by 5th May 2025

Analysis S1: Analysis of health insurance service data

Background and Method

While the DHS provided individual-level NHIP enrolment data, it lacked district-level insights on actual enrolment rates and distribution of empaneled health facilities. To address this gap, we constructed a supplementary dataset for districts and provinces by merging three data sources: (1) real-time NHIP enrolment data by district/province from the HIB dashboard till June 2025 (Health Insurance Board 2025a), (2) district/province level data of number of empaneled health facilities from HIB till June 2025 (Health Insurance Board 2025b) and (3) district/province level population data from recent census 2021 (National Statistics Office 2024). This additional dataset provided information about enrolment (%) and health facility density (number of NHIP empaneled health facility per 100,000 population (S1 File Table S4).

We used this additional data to understand the barriers like distribution of NHIP-affiliated health facilities by district and province (Table S5) and its impact in enrolment in NHIP. By correlating NHIP enrolment with health facility density, we assessed whether low enrolment in district level was driven by inadequate access to NHIP service providers.

This approach of using supplementary data provided a more nuanced assessment of intermediatory inequalities NHIP enrolment disparities, informing evidence-based policy to strengthen NHIP equity in Nepal.

Results and Discussion

Figure S2 below shows geographic disparity to NHIP enrolment by district and province. The findings show that Koshi has highest enrolment (52%) while Madhesh province has lowest level of enrolment (12%). Going through the district level, Palpa(99.2%), Jumla (79.2%) Chitwan (79.0%), Jhapa (76.8%), and Bhaktapur (65.1%) are top five performing districts while Sarlahi (6.8%), Dhanusha (7.9%), Mahottari (8.7%), Dang (10.4%), and Bara (11.4%) are the least-five performing districts. Higher enrolments in districts like Palpa, Chitwan, and Jumla may be partially linked to major empanelled tertiary-level facilities, such as Lumbini Medical College in Palpa, Chitwan Medical College in Chitwan, and Karnali Academy of Health Sciences in Jumla district. Other districts like Dhanusha, Banke, and Dang also have similar tertiary level of healthcare which suggest inconsistencies with our previous arguments.

Similarly, province-level analysis showed significant disparities, with Koshi (51.9%) and Gandaki (48.8%) showing higher enrolment while Madhesh (11.9%) and Sudurpaschim (23.2%) provinces had lowest enrolment. These findings demonstrate consistent subnational inequalities across both district and provincial levels, highlighting stark service enrolment gaps in NHIP.

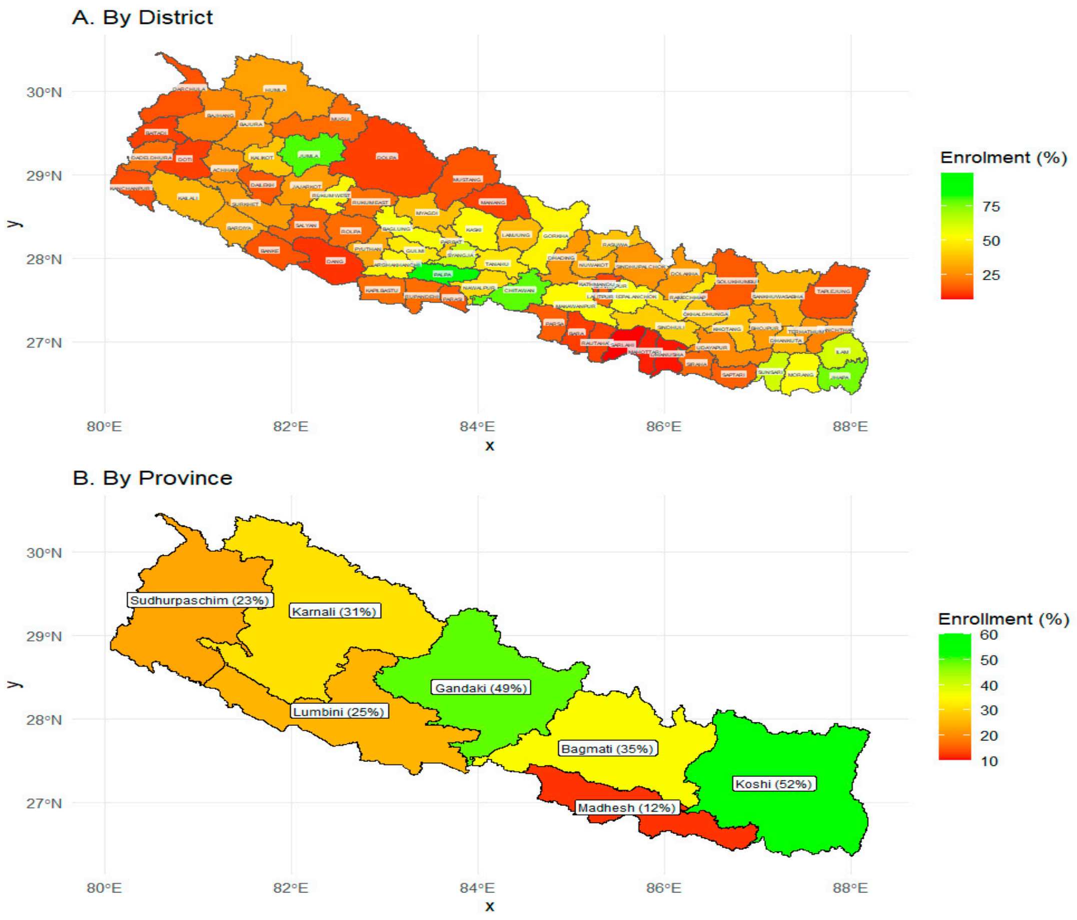

Figure S2: Enrolment in National Health Insurance Program by districts and provinces

We further examined district- and province-level enrolment patterns in relation to the density of NHIP-empanelled health facilities per 100,000 population which is shown in Figure S3. At the district level, nine districts demonstrated particularly low facility density, with fewer than one NHIP service provider density. These included Sarlahi (0.70), Kanchanpur (0.78), Bara (0.79), Rautahat (0.86), Kapilvastu (0.88), Siraha (0.95), Rupandehi (0.98), Mahottari (0.99), and Saptari (0.99). In contrast, districts such as Manang (17.67), Mustang (13.84), and Tehrathum (5.64) showed significantly higher service availability, with facility density of more than five.

Provincial patterns mirror this disparity; Madhesh (0.96) lags far behind Karnali (2.13), Koshi (2.26), and Gandaki (2.43). Notably, six out of Madhesh's eight districts rank among the nine worse-served districts (<1 NHIP facility per 100,000 population). Moreover, four (Sarhahi, Dhanusha, Mahottari and Bara) out of five districts with lowest enrolment rate were from Madhesh province. This spatial overlap suggests lower number empanelled health facilities could have been constrained in NHIP enrolment in Madhesh province.

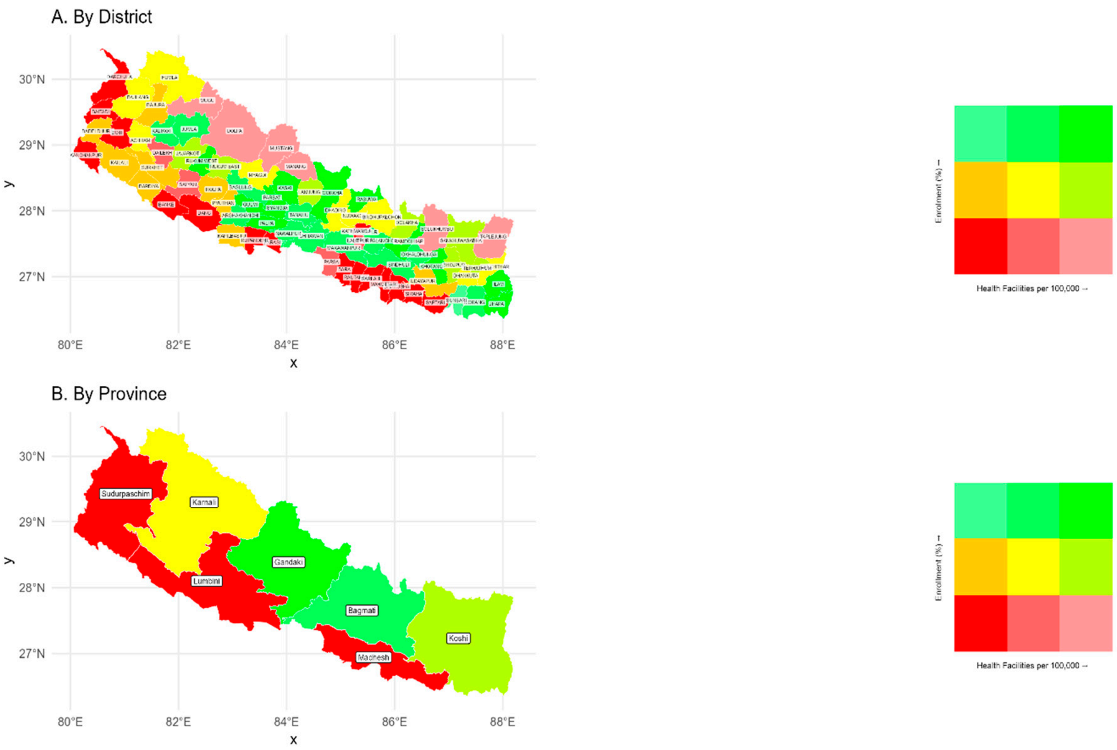

Figure S3: Enrolment percentage and health facility density by districts and province levels

References:

Health Insurance Board (2025a) *Health Insurance Board Dashboard*, Kathmandu, Nepal, https://eservice.hib.gov.np/dashboard/, accessed 5 May 2025.

—— (2025b) 'Service Provider Health Institutions', https://hib.gov.np/en/pages/health-care-providers, accessed 5 May 2025.

National Statistics Office (2024) *Nepal Statical Yearbook 2023*, Kathmandu, Nepal, https://giwmscdntwo.gov.np/media/pdf_upload/Nepal%20Statistical%20Year%20book%202023_e3ovoqz.pdf, accessed 5 May 2025.
